# Longitudinal Study of Transmission in Households with Visceral Leishmaniasis, Asymptomatic Infections and PKDL in Highly Endemic Villages in Bihar, India

**DOI:** 10.1371/journal.pntd.0005196

**Published:** 2016-12-14

**Authors:** Vidya Nand Ravi Das, Ravindra Nath Pandey, Niyamat Ali Siddiqui, Lloyd A C Chapman, Vijay Kumar, Krishna Pandey, Greg Matlashewski, Pradeep Das

**Affiliations:** 1 Rajendra Memorial Research Institute of Medical Sciences (ICMR), Patna, India; 2 University of Warwick, Coventry, England; 3 Department of Microbiology and Immunology, McGill University, Montreal, Quebec, Canada; Institut Pasteur, FRANCE

## Abstract

**Background:**

Visceral Leishmaniasis (VL) is a neglected tropical disease that afflicts some of the poorest populations in the world including people living in the Bihar state of India. Due to efforts from local governments, NGOs and international organizations, the number of VL cases has declined in recent years. Despite this progress, the reservoir for transmission remains to be clearly defined since it is unknown what role post kala-azar dermal leishmaniasis (PKDL) and asymptomatic infections play in transmission. This information is vital to establish effective surveillance and monitoring to sustainably eliminate VL.

**Methodology/Principal Findings:**

We performed a longitudinal study over a 24-month period to examine VL transmission and seroconversion in households with VL, PKDL and asymptomatic infections in the Saran and Muzaffarpur districts of Bihar. During the initial screening of 5,144 people in 16 highly endemic villages, 195 cases of recently treated VL, 116 healthy rK39 positive cases and 31 PKDL cases were identified. Approximately half of the rK39-positive healthy cases identified during the initial 6-month screening period were from households (HHs) where a VL case had been identified. During the 18-month follow-up period, seroconversion of family members in the HHs with VL cases, PKDL cases, and rK39-positive individuals was similar to control HHs. Therefore, seroconversion was highest in HHs closest to the time of VL disease of a household member and there was no evidence of higher transmission in households with PKDL or healthy rK39-positive HHs. Moreover, within the PKDL HHs, (the initial 31 PKDL cases plus an additional 66 PKDL cases), there were no cases of VL identified during the initial screen or the 18-month follow-up. Notably, 23% of the PKDL cases had no prior history of VL suggesting that infection resulting directly in PKDL is more common than previously estimated.

**Conclusions/Significance:**

These observations argue that acute VL cases represent the major reservoir for transmission in these villages and early identification and treatment of VL cases should remain a priority for VL elimination. We were unable to obtain evidence that transmission occurs in HHs with a PKDL case.

## Author Summary

Visceral leishmaniasis (also known as kala-azar) caused by infection with *L*. *donovani* is a deadly parasitic disease that afflicts some of world’s poorest populations, including the people of the northern Bihar State of India. Once transmitted to a human by an infected sandfly, the *L*. *donovani* parasite migrates from the site of the sandfly bite throughout the reticuloendothelial system, resulting in high levels of infection in the spleen, liver and bone marrow that eventually lead to organ failure and death if not treated effectively. India, Nepal and Bangladesh are currently engaged in a program to eliminate visceral leishmaniasis, principally through early case detection, treatment and vector control. As humans are the only reservoir for *L*. *donovani*, it is necessary to understand how the disease is transmitted and specifically what role acute visceral leishmaniasis (VL) cases, asymptomatic infections and post kala-azar dermal leishmaniasis (PKDL) cases play in transmission. We therefore performed a study to determine seroconversion for antibodies against the *L*. *donovani* rK39 antigen as a surrogate for transmission in households with VL cases, asymptomatic infections and PKDL cases in 16 highly endemic villages over a 2-year period in Bihar, India. We observed that most transmission occurred in the VL households and further that it occurred closest to the time of acute disease. We were unable to confirm that transmission occurred in the households with either asymptomatic infections or PKDL cases. These observations argue that active surveillance to diagnose and treat VL cases as soon as possible to reduce transmission should remain a priority for VL elimination.

## Introduction

Visceral leishmaniasis (VL), also known as kala-azar, is a neglected vector-borne disease caused by a protozoan parasite, *Leishmania donovani* and is transmitted by the bite of infected *Phlebotomus argentipes* sandflies. The estimated number of annual VL cases worldwide is 0.2–0.4 million per year, and the majority of the cases occur in India, Bangladesh, Sudan, Ethiopia and Brazil [1]. India alone contributes up to 50% of the worldwide VL cases [1], and 80% of these cases are from the northern Bihar State [2]. Most of the endemic population are from rural areas living in mud adobe houses [3]. During the 2005 world health assembly, the governments of India, Nepal and Bangladesh committed to eliminate VL with a target of less than 1 case per 10,000 in all highly endemic regions by 2015 [4]. Although this date has been extended, significant progress has been made largely due to the availability of point of care diagnostics and effective treatments at the primary health care (PHC) level. However, this target will likely not be met in the near future in the Bihar state of India, which continues to have the highest number of cases.

One of the challenges of eliminating VL is that not everyone who becomes infected manifests with clinical disease. The role of asymptomatic infections and post kala-azar dermal leishmaniasis (PKDL) in transmission and maintenance of the human reservoir remains poorly understood. Asymptomatic infections include a positive result from serological assays, polymerase chain reaction (PCR), leishmanin skin test (LST) or IFN-γ release assay (IGRA) [5, 6]. It is difficult to know how many asymptomatic carriers there are in the Indian subcontinent because of the different study designs and assays used to identify asymptomatic carriers, but it has been estimated to be in the range of 5–10 asymptomatic cases for each active VL case [5, 6, 7]. The immunological, nutritional, environmental and genetic factors that determine progression or protection against VL in asymptomatic cases of *L*. *donovani* infection are unknown.

PKDL usually appears several months after successful treatment of VL, but can more rarely also occur in the absence of prior VL [8, 9]. PKDL cases are healthy but display a spectrum of skin lesions ranging from hypo-pigmented macules to papules and nodules over the body and face, which can be mistaken for other skin conditions including leprosy [8, 9]. In the Indian subcontinent, PKDL occurs in about 5–10% of cured VL patients and in Sudan it occurs in up to 50% of cured patients. It has been reported that PKDL represents a reservoir capable of starting new epidemics of VL [10], however this requires verification and more study. Since PKDL does not cause clinical discomfort, patients rarely seek treatment.

Only a few longitudinal studies have been performed and these have shown that living in the same household (HH) with a VL case strongly predisposes individuals to developing VL [11, 12]. However, several aspects of VL transmission and development require investigation, including the role of PKDL and asymptomatic infections in transmission. The assumption of this study was that transmission occurs in VL and PKDL HHs, but the relative levels of transmission between these reservoirs remains to be determined. The contribution asymptomatic infections have on transmission is not known. Understanding the relative contributions of *L*. *donovani* transmission by individuals with PKDL and asymptomatic infections is crucial for designing control and elimination strategies [13, 14]. To address these issues, a longitudinal study was undertaken to follow family members from households with asymptomatic and PKDL cases alongside households with VL cases. Transmission to family members in these households was determined by monitoring the development of new VL cases and conversion to rK39-positivity over a period of 24 months.

Since this was a longitudinal study involving thousands of individuals, it was necessary to use an assay acceptable to the population and which could be performed routinely in the field. The rK39 rapid diagnostic test (RDT), which detects the presence of antibodies against the K39 *L*. *donovani* antigen, was therefore used. Although this assay is not sensitive enough to identify all asymptomatic infected cases, it does have the same sensitivity and specificity for each group and thus provides an accurate comparison of relative seroconversion between groups. Individuals with higher rK39 or DAT antibody titers have been reported to be associated with higher conversion rates to VL than people with low antibody titers [15,16,17]. We therefore believe that the rK39 RDT was an appropriate assay for comparing transmission between different cohorts in this longitudinal study.

## Methods

### Sample size

Our primary aim in conducting the study was to test the hypothesis that there is greater transmission in households (HH) in which there are VL cases than in HH with healthy rK39-positive individuals or HH with only PKDL cases. Cumulative incidence of rK39 seroconversion in each of the cohorts over the course of the study was used as the measure of transmission. Sample size was calculated based on the assumption that at least 6% of the population in the highly endemic regions would become asymptomatically infected and seroconvert over 2 years [7,16,18]. In order to detect an odds ratio of 3.0 in the difference in seroconversion risk between any pair of cohorts, with a significance of 0.05 and a power of 0.8, at least 151 people were required in each cohort.

### Selection of endemic villages and screening

Sixteen highly endemic villages from the Paroo, Sahebganj, Baniyapur and Marhoura blocks were selected on the basis of having the highest number of VL cases in 2012 and 2013 in the highly endemic Saran and Muzaffarpur districts in the state of Bihar. Using medical records from the primary health centers (PHC) to identify previously treated VL cases and corresponding village addresses, a survey was conducted by field technicians in the selected villages to obtain details from all VL affected HHs including details of illness, diagnosis, treatment and affected family members using a structured questionnaire. Within the 16 villages, 1442 HH were in the highly endemic area and 203 HH were removed from the study since they had VL cases 2 years previous to the study (2011 or earlier). Of the 1239 HH remaining, the population was determined to be 8805 of which 5144 were tested with the rK39 RDT since not everyone was available during the serology camps and furthermore, we restricted our screening activity to the endemic clusters of the villages. Inclusion criteria were all individuals living in the endemic villages. The only exclusion criteria included members from HHs with VL cases 2 years prior to the study since we were unsure what effect this could have on transmission and children below the age one year old. Serology was also performed in the endemic villages with the rK39 (InBios Inc.) rapid diagnostic test (RDT). Baseline data on VL cases, rK39 serology, and PKDL incidence was obtained from April—October, 2013. Follow-up in the same endemic villages was performed every 6 months for 18 months up to April 2015 to identify new VL and PKDL cases, and to repeat serological testing with the rK39 RDT. Conversion to rK39-positive was considered the criteria for transmission of *L*. *donovani*. VL cases were identified as having fever for more than 2 weeks, splenomegaly and a positive test on the rK39 RDT. PKDL cases were identified using the rK39 RDT and microscopy on skin biopsies. The rK39 RDT was performed on a finger-prick drop of blood according to the manufacturer’s (InBios Inc.) instructions.

One hundred and fifty-two healthy control HHs from the same endemic villages where the entire family were healthy and rK39-negative were also selected for the 18-month follow-up period. Four cohorts of HHs were identified: VL, PKDL, healthy rK39-positive, and control. Retrospective studies of an additional 49 PKDL cases were also carried out on cases admitted and treated in 2012 and 2013 at the Rajendra Memorial Research Institute of Medical Sciences (RMRIMS) and on 17 PKDL cases identified beyond the 16 study villages.

### Data collection, monitoring and analysis

A written register was maintained in each participating PHC containing the details of HH members and addresses, and the rK39 test strips were attached to the register for display against the names of VL, PKDL, rK39+ healthy and control cases. Each of the 4 PHCs in the Paroo, Sahebganj, Baniyapur and Marhoura blocks had 2 technicians and one field surveyor to facilitate and carry out the screening activities in the villages. The project team of RMRIMS undertook regular monitoring of rK39 RDT screening activities in the selected endemic villages. The role of the project monitoring team was to supervise the screening activities and provide technical guidance. During the field monitoring activities, health camps were routinely conducted in the endemic villages where local villagers were examined and treated by the clinician of the project team for minor illness and distribution of basic medicines including analgesic, anti-diarrheal tablets, iron supplements, cough syrup and de-worming treatment. Treatments were the same for each cohort. The health camps were necessary to obtain the confidence of the rural population for repeatedly screening with the rK39 RDT and at the same time provided relief from common illness. During the field monitoring, VL cases under treatment were also followed and given necessary advice to complete the treatment.

The data generated in the field was doubly entered in the software Epi Info version 3.5.1, specifically designed for the study independently by two Data Entry Operators. Upon completion of the 2^nd^ entry, the two files were compared. In case of discrepancies, corrections were made after reviewing the original questionnaire and data entry forms. The online software, GraphPad QuickCals was used for data analysis. Fisher’s Exact two-tailed tests were performed to determine whether there were significant differences between the different cohorts. Odds ratios for family members converting to rK39-positive were calculated for the different cohorts (VL, PKDL and healthy rK39-positive households) for follow-up months 6–18 using online MedCalc statistical software.

### Ethical considerations

The study was approved by the ethics committee of Rajendra Memorial Research Institute of Medical Sciences (RMRIMS), Agamkuan, Patna, India. Before the start of screening activities, written informed consent was obtained for participation in the study and issues regarding confidentiality, publication and approval of participation obtained by signature or thumb impression in the presence of a witness.

## Results

**Fig 1:**
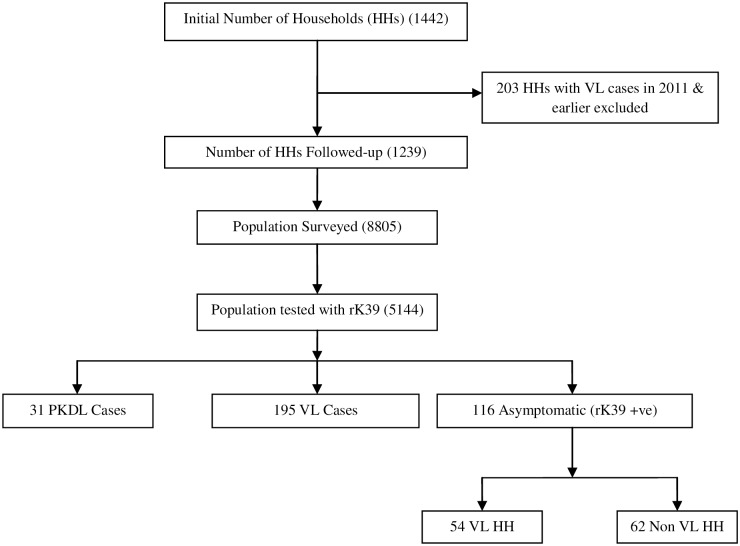
Outline of the different cohorts identified during the initial screening.

Sixteen highly endemic villages were selected in the Muzaffarpur and Saran districts of Bihar on the basis of clinical record information from local PHCs revealing these villages to be highly endemic for VL. Household (HH) heads were interviewed and the village populations tested with the rK39 RDT to detect the presence of antibodies against the K39 *L*. *donovani* antigen. At the study baseline, 5144 individuals were tested with the rK39 RDT from 1442 HH within the 16 selected villages in the Muzaffarpur and Saran districts of Bihar. Fig 1 outlines the different cohorts identified, including VL, PKDL and healthy rK39-positive individuals. The age and sex of the different cohorts is summarized in Table 1. The incidence of VL cases from April—October 2013, calculated as a percentage of the screened population that had VL, was similar in each age category (there were no statistically significant differences). The age incidence distribution was similar in the healthy rK39-positive group, but with lower incidence in the 16-30yr age group (p < 0.0001). In the PKDL group, there were no statistically significant differences in incidence between age groups.

**Table 1:** Demographic Characteristics of Cohorts at the Baseline Survey.

Characteristics and age group (yrs)	Screened Population^1^	No. Male	No. Female	Total (incidence,%)^2^	p-value^3^
**Visceral Leishmaniasis cases**					
1–15	2283	45	39	84 (3.68%)	0.36
16–30	1136	15	14	29 (2.55%)	0.026
31–45	817	25	17	42 (5.14%)	0.50
≥ 46	908	24	16	40 (4.41%)	Reference
Total	5144	109	86	195 (3.79%)	
**Healthy rK39 –positive cases**					
1–15	2283	16	34	50 (2.19%)	0.035
16–30	1136	4	7	11 (0.97%)	<0.0001
31–45	817	10	13	23 (2.82%)	0.41
≥ 46	908	18	14	32 (3.52%)	Reference
Total	5144	48	68	116 (2.26%)	
**PKDL cases**					
1–15	2283	6	11	17 (0.74%)	0.22
16–30	1136	2	5	7 (0.62%)	0.53
31–45	817	2	2	4 (0.49%)	0.71
≥ 46	908	3	0	3 (0.33%)	Reference
Total	5144	13	18	31 (0.60%)	

^1^. A total of 5144 rK39 RDTs were performed from 1239 HH in 16 highly endemic villages.

^2^. Incidence calculated as the percentage of the screened population in each age group who had VL, were rK39+ or had PKDL from 04/2013–10/2013.

^3^. p-values calculated using Fisher’s Exact two-tailed test. Significance determined at p < 0.05/3 = 0.017 using the Bonferroni correction to account for multiple comparisons.

From the initial baseline screening, VL, PKDL and healthy rK39-positive cases were identified (Table 2). There were 195 cases of VL where 170 HH had one case, 11 HH had 2 cases and 1 HH had 3 cases. There were also 54 healthy rK39-positive cases identified in the VL HHs. There were 31 PKDL cases identified in 31 HHs in which no other family members had VL, PKDL or were rK39-positive. There were 116 healthy rK39-positive cases in which 54 were from the VL HHs and 62 were from non-VL HHs. The fact that almost half of the rK39-positive cases were from VL HH (54/886 RDTs from VL HHs versus 62/4258 RDTs from non-VL HH), this demonstrates that transmission was more likely to occur in the VL HHs than in the non-VL HHs (Odds ratio = 4.39, 95% confidence interval = 3.03–6.37, z statistic = 7.8, p < 0.0001).

**Table 2:** Number of Cases Identified in Initial Screening (Months 0–6) in Households with VL, PKDL and rK39-positive.

Household Category	No. of rK39 RDTs performed on family members ^2^	No. VL cases^3^	No. rK39 positive healthy cases	No. PKDL cases
VL	886	195	54	0
rK39-positive^1^	261	0	62 ^4^	0
PKDL^5^	153	0	0	31
Healthy	3844	0	0	0

^1^. Includes rK39-positive cases outside of VL HHs

^2^. A total of 5144 rK39 RDTs were performed from 1239 HH in 16 highly endemic villages.

^3^. From 182HH where 170 HH had 1 case of VL, 11 HH had 2 cases of VL, 1 HH had 3 cases of VL and 47 VL HH had 54 healthy rK39-positive cases.

^4^. 49 HH had 1 case of rK39 (+) healthy, 5 HH had 2 cases of rK39 (+) healthy, 1 HH had 3 cases of rK39 (+) healthy.

^5^. None of the PKDL HH had VL or rK39 (+) healthy cases.

Household members from the VL, PKDL and asymptomatic rK39-positive cohort HHs were then followed prospectively for 18 months to determine how many converted to rK39-positive (Table 3). For this analysis, 692 individuals from 152 HH living in close proximity to the VL HHs were selected as controls where all family members were healthy and rK39-negative. An interesting observation from the 18-month follow-up was that the number of new conversions to rK39-positive was relatively few in the VL HHs (24 cases); [8(at 6 months) +13(at 12 months) +3(at 18 months) = 24] compared to the initial screen at 0 months (54 cases). This is likely because the VL cases had all received treatment and thus become less infectious to the sandfly vector resulting in less transmission to family members during the 18-month follow-up. Seroconversion in the VL HHs was similar to the control HHs [10(6 months) + 4(12 months) + 2(18 months) = 16 cases in control HH] (p = 0.6332 for a test of difference in seroconversion incidence). Seroconversion to rK39-positive during the 18-month follow-up was also low in the HHs with existing healthy rK39-positive cases (2 cases) and the PKDL HHs (2 cases) and was less than in the control HH. In the VL HHs, 34 of the 54 rK39-positive healthy cases became rK39-negative and one developed PKDL during the 18-month follow-up. In the healthy rK39-positive HHs, 36 of the 62 rK39-positive cases became rK39-negative, one developed VL and one developed PKDL during the 18-month follow-up.

**Table 3:** Transmission: New rK39-positive Cases during the 6–18 Month follow-up.

Baseline rK39 positive cases			New rK39 positive cases identified						
Category / No. Households (HHs)	0 Months (Baseline)		6 Months (follow-up)		12 Months (follow-up)		18 Months (follow-up)		Transmission^5^6–18 month No. positive/No. tests (%)
	No.^1^ Tests	No. posit	No. Tests	No. Posit	No. Tests	No. Posit	No. Tests	No. Posit	
VL / 182 HHs	886	54 ^2^	1024	8	1051	13	1007	3	24/3082 (0.78)
Healthy rK39+ / 55 HHs	261	62 ^3^	316	0	314	1	309	1	2/939 (0.21)
PKDL / 31 HHs	153	0	187	0	161	0	167	2	2/515 (0.39)
Control^6^ / 152 HHs	692	NA^4^	859	10	845	4	783	2	16/2487 (0.64)

^1^. Indicates number of rK39 RDTs performed on family members for each cohort.

^2^. There were 54 healthy rK39-positive cases of whom one developed PKDL and 34 became seronegative during the 18 month follow-up.

^3^. There were 62 healthy rK39-positive cases of whom one developed VL, one developed PKDL, and 36 became seronegative during the 18 month follow-up period.

^4^. NA: not applicable since these HH were selected to be all rK39-negative at baseline.

^5^. Indicates number of new positives/total number of rK39 RDTs for the 6 to 18 month follow-up.

^6^. Control HHs contained rK39-negative healthy individuals.

Overall, these observations show no significant increased transmission to new rK39-positive cases in the VL, rK39-positive and PKDL cohorts compared to the control cohort during the 6–18 month follow-up. This suggests that most seroconversion in family members occurred closest to the time of disease during the initial screening period (Table 2).

Considering the above observations, we were interested to further examine transmission in PKDL HHs since it has been generally held that PKDL HHs represent an important reservoir for transmission of VL [10]. We therefore identified an additional 66 cases of PKDL and performed rK39 RDTs on all of their family members, and also determined whether any of their family members developed VL or PKDL over an 18-month period. We further stratified these additional 66 PKDL cases and the original 31 cases (from Table 1) according to clinical presentation, including whether the lesions were macular, papular or nodular. As shown in Table 4, about 60% of the cases were macular followed by about 30% mixed and about 10% nodular. It is noteworthy that there were no cases of VL among the HH family members living with the 97 PKDL cases at baseline or follow-up. There were only 3 cases of individuals becoming rK39-positive in which 2 were from the 18-month follow-up performed on the original 31 PKDL cases identified in Table 4. Considering the entire PKDL cohort, the risk of conversion to rK39-positive was not greater than in the control group in Table 3.

**Table 4:** VL and rK39 serology in Family Members of PKDL Households.

Lesion Type	Number	Transmission over 18 Months	
		VL	rK39 (+)
Macular	58	0	2
Papular	2	0	0
Nodular	9	0	0
Mixed	28	0	1
Total	97 (31+17+49) ^1^	0	3

^1^. The 31 PKDL cases were from the original screening of the 16 villages described above, 17 cases were identified beyond the 16 villages and 49 cases were recently admitted and treated at RMRIMS and their family members were followed prospectively as described above.

There appears to be an increased number of PKDL cases treated in Bihar since currently there are more patients treated for PKDL than for VL at RMRIMS. We therefore also determined how many of the PKDL cases were in fact post kala-azar or developed PKDL without prior treatment of VL. A total of 23% were not previously treated for VL revealing a substantial number of PKDL cases developed from asymptomatic cases without conversion to VL. Therefore, the development of PKDL without VL is relatively common in these communities.

## Discussion

The reservoir for VL transmission in South East Asia remains poorly understood and this is hampering the development of strategies for sustainable VL elimination. The objective of this longitudinal study was to compare households with VL, PKDL and healthy rK39-positive cases with respect to family members developing VL or becoming rK39-positive. The major observation was that transmission to rK39-positive in family members occurs predominantly in VL HHs and that transmission occurs close to the time of disease, presumably when parasite levels are highest prior to treatment. Seroconversion in VL HHs after treatment (6–18 month follow-up), in rK39-positive healthy HHs and in PKDL HHs was similar to control HHs. This was somewhat surprising since it is widely believed that PKDL represents an important reservoir for transmission [10]. Similarly, transmission in a HH with a healthy rK39-positive individual appears to be relatively minor as only 7 out of 55 HH had more than one rK39-positive individual during the initial screen and there were relatively few (only 2) new rK39-positive cases identified during the 18-month follow-up in these HHs. The low level of transmission in HH with a healthy rK39-positive individual compared to HH with a VL case could be due to the much lower level of parasite in the blood of asymptomatically infected individuals. It has been reported that asymptomatic individuals have 0.01–5 parasites per ml of blood compared to 8–50,000 parasites ml of blood in VL cases [19]. Considering that a sandfly blood meal is about 10 ul; this would represent less than a single parasite from the asymptomatic cases with the highest level of parasitemia. This low level of parasite in the asymptomatic cases [19] indicates that these individuals have developed immunity to control parasite levels and it will be important to understand how tolerance develops in these individuals. Similar villages as identified in this study could be useful for such studies and perhaps identify in advance which villages are vulnerable to future outbreaks.

During the follow-up period, over half (70/116) of the healthy rK39-positive cases became rK39-negative, 1 developed VL and 2 developed PKDL. These observations are consistent with a previous study reporting that within one year about 80% of healthy rK39-positive cases became seronegative [16] and one out of 50 direct agglutination test (DAT)-positive cases developed VL [7].

In Nepal, it has been reported that about 5% of treated VL patients subsequently develop PKDL [20] whereas in Bangladesh and India the conversion to PKDL is about 10% [21, 22]. We observed that about 23% of PKDL cases had not previously been diagnosed with VL and this is consistent with a previous study reporting that 18% of PKDL cases had no prior history of VL [23]. These observations bring into question whether the term ‘post kala-azar dermal leishmaniasis’ is accurate since a significant number of PKDL cases are not associated with prior kala-azar. It is unclear what evolutionary advantage there is for *L*. *donovani* to cause PKDL since the observations from this study suggest that PKDL does not appear to provide a major reservoir for transmission. Alternatively, PKDL may arise as a result of the host protective immune response in some individuals.

Taken together, the observations from this study fail to provide evidence that PKDL represents a risk factor for development of VL or becoming asymptomatic rK39-positive. It will be necessary to follow a larger cohort of family members of PKDL cases with nodular lesions to determine if nodular cases transmit, since there are a larger number a parasites in the nodules than in macular lesions [22]. Even though PKDL may not be a major source of disease transmission, it may nevertheless represent a potential reservoir that maintains the parasite in these endemic regions for long periods and in some instances could be a source of infection in sandflies [10]. Based on these observations, consideration should be given to the management of PKDL, including whether treatment is justified considering that current therapies are highly toxic and have low compliance.

It is important to appreciate that rK39-positive seroconversion is only one criteria to identify asymptomatic infections with *L*. *donovani* and was used in this investigation as a tool to study transmission and not to identify all of the asymptomatic cases in the endemic villages. Other assays such as the IFN-γ release assay (IGRA), leishmanin skin test (LST), DAT or PCR will identify more cases. Currently, there is no universal definition of an asymptomatic *L*. *donovani* infected individual. Moreover, there is no simple assay to measure parasite load in VL or asymptomatic infections, which would help to identify the *L*. *donovani* reservoir more precisely and perhaps identify those asymptomatic cases at risk of progression to disease. Xenodiagnosis and sandfly infection studies involving households with VL, PKDL and rK39-positive cases could also help establish a better understanding of the reservoir for transmission of *L*. *donovani* to the sandfly vector and maintenance of the *L*. *donovani* reservoir in Bihar.

It has been reported that a higher antibody titer in asymptomatic cases is associated with a greater likelihood of progression to VL [15,16,17]. Since the rK39 RDT requires a higher titer to be positive than an rK39 ELISA [24], this would suggest that the rK39 RDT could identify those with higher antibody titers and therefore greater risk of progression to VL. However, we identified only one case of VL developing in a healthy rK39-positive individual over the 18-month follow-up period. This suggests that seroconversion using the rK39 RDT may not provide a strong prognostic indication of VL.

It is noteworthy that once a cluster of VL cases in an endemic village is identified for study, the local epidemic is already at the tail end, which makes longitudinal studies such as the one reported here difficult to perform. It would be useful in future to perform the rK39 RDT on family members and surrounding HH as soon as a VL case reports to a PHC for treatment. This could establish a cohort at the beginning of a local epidemic and could also help to define a potential cohort for future vaccine trials.

Although these observations provide insight into VL transmission in highly endemic villages, further studies are needed to understand how the parasite reservoir is maintained in areas where there are very few VL cases, such as in the hilly regions of Nepal and Bhutan [25, 26], and why VL cases tend to cluster and occur in shifting and tightly localized areas. With respect to control measures, the results from this study argue that every effort should be made to identify and treat acute VL cases as soon as possible, since this appears to be the major source of transmission. This is consistent with a recent study involving mathematical modeling which likewise concluded that shortening the time to diagnose and treat VL cases would result in a dramatic reduction in the incidence of new VL cases [27]. One approach to improve surveillance of VL among large rural populations would be to expand and strengthen the training of village accredited social health activists (ASHA) to identify VL cases in endemic villages and ensure treatment at the local primary health care centers [28, 29]. This will require continuing strong commitment from NGOs, and local and national governments.

### Ethics statement

The study was approved by the ethics committee of Rajendra Memorial Research Institute of Medical Sciences (RMRIMS)

## Supporting Information

S1 Checklist STROBE checklist. (PDF)

## References

[1] Alvar J, Velez ID, Bern C, Herrero M, Desjeux P, Cano J, Jannin J, Boer Md, the WHO Leishmaniasis Control Team. Leishmaniasis worldwide and global estimates of its incidence. PloS One 2012; 7(5):e35671 10.1371/journal.pone.0035671 22693548 PMC3365071

[2] National Vector Borne Disease Control Programme, Directorate General of Health Services, Ministry of Health and Family Welfare, Government of India. Kala-azar Cases and Deaths in the Country since 2010. http://nvbdcp.gov.in/ka-cd.html. Accessed June 2016.

[3] Boelaert M, Meheus F, Sanchez A, Singh S, Vanlerberghe V, Picado A, Meessen B, and Sundar S. The poorest of the poor: a poverty appraisal of households affected by visceral leishmaniasis in Bihar, India. Trop Med Internal Health 2009; 14: 639–644. 10.1111/j.1365-3156.2009.02279.x 19392741

[4] World Health Organization. Regional strategic framework for elimination of kala-azar from the South-East Asia region (2005–2015). New Delhi: WHO Regional Office for South-East Asia, 2005.

[5] Das S, Matlashewski G, Bhunia G, Kesari S, and Das P. Asymptomatic Leishmania infections in northern India; a threat for the elimination program? Trans R Soc Trop Med Hyg 2014; 679–684, 2014. 10.1093/trstmh/tru146 25205664

[6] Singh O, Hasker E, Sacks D, Boelaert M, and Sundar S. Asymptomatic Leishmania infection: A new challenge for Leishmania Control. Clin. Infect. Dis. 2014; 58:1424–1429. 10.1093/cid/ciu102 24585564 PMC4001287

[7] Ostyn B, Gidwani K, Khanal B, Picado A, Chappuis F, Singh SP, Rijal S, Sundar S and Boelaert M. Incidence of symptomatic and asymptomatic *Leishmania donovani* infections in high-endemic foci in India and Nepal: a prospective study. PLoS Neg. Trop. Dis. 2011; 5(10): e1284. 10.1371/journal.pntd.0001284 PMC3186756 21991397

[8] Zijlstra E, Musa A, Khalil E, and El Hassan I. Post-kala-azar dermal leishmaniasis. Lancet Infect Dis 2003; 3: 87–97. 12560194 10.1016/s1473-3099(03)00517-6

[9] Murray HW, Berman JD, Davies CR, and Saravia NG. Advances in leishmaniasis. Lancet 2005; 366: 1561–1577. 10.1016/S0140-6736(05)67629-5 16257344

[10] Addy M and Nandy A. Ten years of post kala-azar dermal leishmaniasis initate the outcome in 24-paragans? Bull World Health Organ 1992; 70: 341–346. 1638662 PMC2393278

[11] Gidwani K, Kumar R, Rai M and Sundar S. Longitudinal seroepidemiologic study of visceral leishmaniasis in hyperendemic regions of Bihar, India. Am J Trop Med Hyg 2009; 80(3): 345–346. 19270279

[12] Bern C, Haque R, Chowdhury R, Ali M, Kurkjian K, Vaz L, Wahed M, Wagatsuma Y, Breiman RF, Williamson J, E, Secor WE, and Maguire JH. The epidemiology of visceral leishmaniasis and asymptomatic Leishmania infection in a highly endemic Bangladeshi village. Am. J Trop Med Hyg 2007; 76(5): 909–914. 17488915

[13] Rock K, Rutte E, de Vlas S, Adams E, Medley G, and Hollingsworth D. Uniting mathematics and biology for control of visceral leishmaniasis. Trends Parasit. 2015; 31(6): 251–259. 10.1016/j.pt.2015.03.007 25913079

[14] Cameron M, Acosta-Serrano A, Bern C, Boelaert M, den Boer M, Burza S, Chapman L. et al Understanding the transmission dynamics of Leishmania donovani to provide robust evidence for interventions to eliminate visceral leishmaniasis in Bihar, India. Parasit and Vector 2016; 9(25) 10.1186/s13071-016-1309-8 PMC4729074 26812963

[15] Hasker E, Malaviya P, Gidwani K, Picado A, Ostyn B, Kansal S, Singh RP, Singh OP, Chourasia A, Singh AK, Shankar R, Wilson ME, Khanal B, Rijal S, Boelaert M, Sundar S. Strong association between serological status and probability of progression to clinical visceral leishmaniasis in prospective cohort studies in India and Nepal. PLoS Negl Trop Dis 2014; 8(1): e2657 10.1371/journal.pntd.0002657 24466361 PMC3900391

[16] Hasker E, Kansal S, Malaviya P, Gidwani K, Picado A, Singh RP, Chourasia A, Singh AK, Shankar R, Menten J, Wilson ME, Boelaert M, Sundar S. Latent infection with *Leishmania donovani* in highly endemic villages in Bihar, India. PLoS Negl Trop Dis 2013; 7(2):e2053 10.1371/journal.pntd.0002053 23459501 PMC3573094

[17] Chapman L, Dyson L, Courtenay O, Chowdhury R, Bern C, Medley G, and Hollingsworth D. Quantification of the natural history of visceral leishmaniasis and consequences for control. Parasit and Vectors 2015; 8: 521–534. 10.1186/s13071-015-1136-3 PMC4618734 26490668

[18] Picado A, Ostyn B, Singh S, Uranw S, Hasker E, Rijal S, Sundar S, Boelaert M, Chappuis F. Risk factors for visceral leishmaniasis and asymptomatic *Leishmania donovani* infection in India and Nepal. PLoS One 2014; 9(1) e87641 10.1371/journal.pone.0087641 24498159 PMC3909193

[19] Sudarshan M, Sundar S. Parasite load estimation by qPCR differentiates between asymptomatic and symptomatic infection in Indian visceral leishmaniasis. Diagn Micro Inf Dis 2014; 80: 40–42. 10.1016/j.diagmicrobio.2014.01.031 PMC4157122 25023070

[20] Uranw S, Ostyn B, Rijal A, Devkota S, Khanal B, Menten J, Boelaert M, Rijal S. Post-kala-azar dermal leishmaniasis in Nepal: A retrospective cohort study (2000–2010). PLoS Negl Trop Dis 2011; 5(12): e1433 10.1371/journal.pntd.0001433 22206030 PMC3243697

[21] Mondal D and Khan M. Recent advances in post-kala-azar dermal leishmanaisis. Curr Opin Infect Dis 2011; 24(5): 418–422. 10.1097/QCO.0b013e32834a8ba1 21885919

[22] Zijlstra EE, Musa AM, et al Post-kala-azar dermal leishmaniasis. Lancet Infect Dis 2003; 3(2): 87–98. 12560194 10.1016/s1473-3099(03)00517-6

[23] Das VNR, Ranjan A, Pandey K, Singh D, Verma N, Das S, Lal CS, Sinha NK, Verma RB, Siddiqui NA and Das P. Clinico-epidemiologic profile of a cohort of post-kala-azar dermal leishmaniasis patients in Bihar, India. Am J Trop Med Hyg 2012; 86(6): 959–961. 10.4269/ajtmh.2012.11-0467 22665600 PMC3366539

[24] Matlashewski G, Das VNR, Pandey K, Singh D, Das S, Ghosh AK, Pandey RN, Das P. Diagnosis of visceral leishmaniasis in Bihar India: Comparison of the rK39 rapid diagnostic test on whole blood versus serum. PLoS Negl Trop Dis 2013; 7: 5e2233. 10.1371/journal.pntd.0002233 PMC3662694 23717700

[25] Ostyn B, Uranw S, Bhattarai NR, Das ML, Rai K, Tersago K, Pokhrel Y, Durnez L, Marasini B, Auwera der GV, Dujardin JC, Coosemans M, Argaw D, Boelaert M, Rijal S. Transmission of Leishmania donovani in the hills of Eastern Nepal, an outbreak investigation in Okhaldhunga and Bhojpur districts. PLoS Negl Trop Dis 2015; 9(8): e0003966 10.1371/journal.pntd.0003966 26252494 PMC4529159

[26] Yangzom T, Cruz I, Bern C, Argaw D, den Boer M, Vele I, Bhattacharya S, Molina R, and Alvar J. Endemic transmission of visceral leishmaniasis in Butan. Am J Trop Med Hyg 2012; 87: 1028–1037. 10.4269/ajtmh.2012.12-0211 23091191 PMC3516070

[27] Medley G, Hollingsworth D, Olliaro P, and Adams E. Health-seeking behaviour, diagnosis and transmission in control of visceral leishmaniasis. Nature 2015; 528: S102–S108. 10.1038/nature16042 26633763

[28] Das VNR, Pandey RN, Pandey K, Singh V, Kumar V, Matlashewski G, Das P. Impact of ASHA Training on Active Case Detection of Visceral Leishmaniasis in Bihar, India. PLoS Negl Trop Dis 2014; 8(5): e2774 10.1371/journal.pntd.0002774 24853122 PMC4031043

[29] Das VNR, Pandey RN, Kumar V, Siddique N, Verma R, Matlashewski G. and Das P. Repeated training of accredited Social Health Activists (ASHAs) for improved detection of visceral leishmaniasis cases in Bihar, India. Pathogen and Global Health 110 (1): 33–35, 2016. 10.1080/20477724.2016.1156902 PMC4870029 27077313

